# The Mechanical Properties of Nanowires

**DOI:** 10.1002/advs.201600332

**Published:** 2017-01-03

**Authors:** Shiliang Wang, Zhiwei Shan, Han Huang

**Affiliations:** ^1^ School of Mechanical and Mining EngineeringThe University of QueenslandAustralia; ^2^ Center for Advancing Materials Performance from the NanoscaleXi'an Jiaotong UniversityChina

**Keywords:** characterization, mechanical properties, nanowires, size effect

## Abstract

Applications of nanowires into future generation nanodevices require a complete understanding of the mechanical properties of the nanowires. A great research effort has been made in the past two decades to understand the deformation physics and mechanical behaviors of nanowires, and to interpret the discrepancies between experimental measurements and theoretical predictions. This review focused on the characterization and understanding of the mechanical properties of nanowires, including elasticity, plasticity, anelasticity and strength. As the results from the previous literature in this area appear inconsistent, a critical evaluation of the characterization techniques and methodologies were presented. In particular, the size effects of nanowires on the mechanical properties and their deformation mechanisms were discussed.

## 1 Introduction

**Figure 1:**
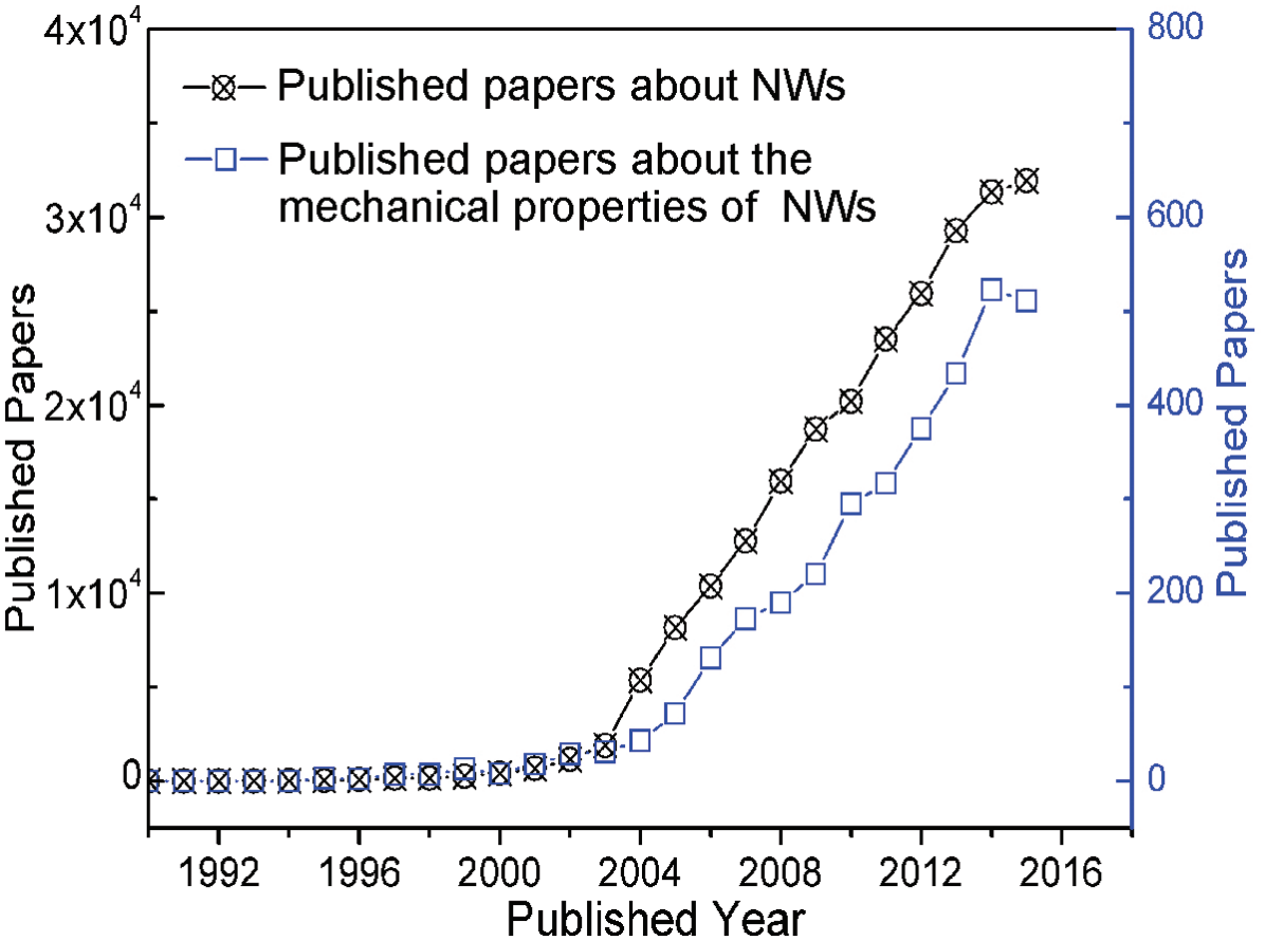
The numbers of published papers on NWs and their mechanical properties, from 1990 to 2015 (extracted from Web−of−Science with the theme words of “nanowire”, “nanowhisker”, “nanorod” or “nanobelt”, and “mechanical property”; http://ipscience.thomsonreuters.com/product/web‐of‐science/(accessed September 2016).

Since its beginnings approximately two decades ago, nanowire (NW) related research has become one of the hottest topics in nanoscience and nanotechnology (**Figure** **1**). The peculiar and fascinating properties of NWs, as well as their unique performance in various applications, have driven tremendous scientific interest.^1^, ^2^ From a practical standpoint, the design, fabrication and application of NW‐based devices^3^ and NW‐strengthened composites^4^ rely critically on the mechanical properties of NWs, which often differ from their bulk counterparts due to their extremely small physical size and high surface‐to‐volume ratio. From a scientific standpoint, NWs provide an ideal test‐bed for understanding the intrinsic and size‐dependent mechanical properties of solid materials, allowing for the validation of theoretical predictions. As a consequence, characterizing and understanding the mechanical properties of NWs has become increasingly important in recent years, as indicated in Figure 1.

The study of the mechanical properties of wire‐like materials at small scale can be dated back to 1924 when Taylor first discovered that microscale metal filaments were highly pliable and had greater tensile strength than wires of ordinary size.^5^ In 1952, Galt and Herring further found that Sn whiskers of ≈ 20 µm diameter exhibited an ultrahigh elastic strain of 2–3%, an order of magnitude higher than their bulk counterparts.^6^ The findings were almost 10% of the theoretical prediction,^7^, ^8^ encouraging the development of whisker fabrication technologies as well as methodologies for characterizing the mechanical property of whiskers.^9^ To date, whiskers of microscale diameters have become one of the most important strengthening materials for fabricating high‐performance composites.^10^ Since 1950s, the diameter‐dependent mechanical properties of whisker materials have been well recognized. However, experimental characterization was limited to whiskers of diameters typically ranging from 1 to 10 µm. The measurement of testing load and strain, as well as the manipulation and gripping of nanoscale specimens, now termed NWs (as also termed nanowhiskers, nanorods, and nanobelts), posed significant challenges. Fortunately, in the past two decades the rapid development of the nano‐manipulation techniques based on atomic force microscope (AFM) and electron microscope (EM),^11^ focus ion beam SEM technology (FIB‐SEM)^12^ and the integration of micro‐electro‐mechanical systems (MEMS) into electron microscopes (EM)^13^, ^14^ has made mechanical characterization of NWs feasible. Research in this area has accelerated rapidly since 2004, and is reflected in the number of published works as shown in Figure 1.

This review focuses on recent developments in the mechanical characterization of NWs over the past 20 years. Widely used methodologies for characterizing the mechanical properties of NWs are reviewed in Section 2, including the introduction of basic strategies, applications and challenges of each method. This is followed by a summary and review of notable findings on the mechanical properties of crystalline NWs, including their elasticity (in Section 3), anelasticity (in Section 4), plasticity (in Section 5) and strength (in Section 6). To conclude, final remarks on the challenges in the current studies are given, and the direction of research in the field as well as future issues are discussed.

## 2 Mechanical Characterization of NWs

A range of techniques have been developed over the past two decades for the mechanical analyses of NWs, with a selection being widely adopted. Methodologies include direct measurements; such as bending, uniaxial loading and indenting; as well as indirect approaches such as vibration. Unlike the mechanical testing of bulk materials, the testing of NWs heavily depends on the experimental setup; in particular, manipulation procedure offers significant challenges due to the small dimensions of the NWs. This section reviews existing mechanical testing methodologies, including the AFM‐based tests, nanoindentation‐based tests, in situ electron microscope (EM) tests and optical microscope based tests, as well as the indirect resonant vibration tests.

### 2.1 AFM‐Based Tests

AFM can measure the force‐displacement response of a small probe in contact with a surface with extremely high resolutions. Therefore, AFM has been a useful tool for measuring the mechanical properties of NWs. Direct AFM‐based measurements such as AFM bending and contact resonance (CR) tests have been widely used for characterizing the mechanical properties of NWs in recent years.

#### 2.1.1 AFM Bending Measurement

**Figure 2:**
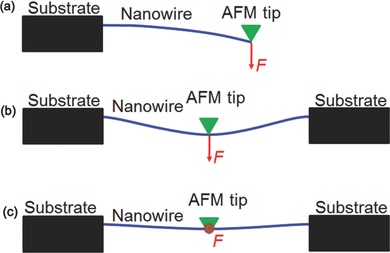
Schematic illustrations of the direct AFM measurements of NWs: a) a NW cantilever with a normal load at the free end; b) a doubly clamped NW loaded normally at the center.

Wong et al. first use the AFM bending technique to measure the mechanical properties of SiC NWs.^15^ In their test, a NW is clamped on a MoS_2_ substrate by a depositing SiO pad to form a cantilever, as shown in **Figure** **2**a. A vertical load is applied to the free end of the cantilever using an AFM tip. By neglecting the contribution of friction force from the substrate of MoS_2_, the modulus, *E*, of SiC NWs (≈600 GPa) can be determined based on the following equation,^15^

(1) $$E=Fl^{3}/\left(3I\delta \right),$$

where ***F*** and ***δ*** are the loading force and corresponding deflection recorded by the AFM, ***l*** is the distance from the loading point to the clamping point, and ***I*** is moment of inertia of the NW. As the friction at NW/substrate interface in ambient atmosphere is usually estimated to be ≈1 MPa,^16^, ^17^, ^18^, ^19^ Kim et al. recently obtained a more sophisticated mechanical model by including the effect of friction from the substrate.^20^ The direct AFM measurement method shown in Figure 2a, was proven to be useful for characterizing the mechanical properties of NWs grown perpendicular on a substrate, such as ZnO NWs^21^ and Si NWs grown on sapphire and Si substrates respectively.^22^ The strain ***ε*** and stress ***σ*** before yielding or fracture can also be derived from the ***F*** − ***δ*** curve obtained from the AFM tip,^23^

(2) $$\varepsilon =3d\delta /2l^{2},$$

(3) $$\sigma =\varepsilon E=3d\delta E/2l^{2},$$

where *d* is the diameter of the NW.

Three‐point bending was first used to measure the mechanical properties of carbon nanotubes by Salvetat et al.^24^ During testing, a nanotube is positioned over a nanopore on a well‐polished alumina ultrafiltration membrane, as shown in Figure 2b. The nanotube, with each end adhered to the substrate surface, can be considered as a doubly clamped beam. Therefore, the modulus of the NW is estimated using the bending‐only model,^24^

(4) $$E=Fa^{3}{\left(L-a\right)}^{3}/\left(3L^{3}I\delta \right),$$

where ***L*** is the suspended length of the nanotube, and *a* is the distance from the loading point to one of the clamped location.

The moduli of different NWs, such as Ag,^25^ CuO^26^ and GaN,^27^ were characterized using an AFM‐based three‐point bending test. In this method a critical condition for achieving measuring accuracy is to ensure sufficiently strong adhesion between the NW and the substrate. Any uncertainty in boundary conditions would have a direct impact on the force‐displacement relationship, leading to large discrepancies (up to four times) in testing.^28^ To avoid uncertainties in the clamped boundary condition, Mai et al. deposited Pt pads at the clamped points of a ZnO nanobelt suspended over a trench on a substrate.^29^ As the deposited pads can provide near‐perfect clamping, the elastic modulus, strain and strain of the doubly clamped NW loaded at the midpoint can be estimated by,^30^

(5) $$\begin{array}{l} E=F_{center}L^{3}/\left(192I\delta_{center}\right),\\ \varepsilon =12\delta_{center}^{2}/5L^{2},\\ \sigma =4F_{center}L/\pi , \end{array}$$

The AFM‐based three‐point bending test has been used for characterizing the elastic modulus of various NWs, including Si,^31^ Ge,^32^ BN,^33^LaB_6_,^34^ TiSi_2_,^30^ NiC,^35^ SiC,^36^ Na_4_Mn_9_O_18_,^37^ SiO_2_,^38^ SrB_2_O_4_,^39^ Co_3_O_4_/CoO,^40^ ZnO,^41^ and metallic glasses.^42^ A sharp AFM tip may cause damage to the NW being tested, as shown in Figure 2b. To prevent such damage occurring, Wu et al. applied load perpendicular to the side surface of a NW, as shown in Figure 2c.^43^

During three‐point bending of a NW, the force‐deflection curves are typically nonlinear, somehow deviated from the behavior defined by Equation (1). This suggests that the presence of axial tension can also significantly affect the *F* − δ relationship. To account for the tension effect, a combined bending‐tension model was developed, in which the elastic modulus is given by,^44^

(6) $$E=F_{cent\mathrm{er}}L^{3}/\left(192I\delta_{cent\mathrm{er}}f\left(\alpha \right)\right),$$

where $(\alpha )=\alpha /(48-192\tanh\left(\sqrt{\alpha }/4)/\sqrt{\alpha }\right)$, and α ≈ 6ε(140 + ε)/(350 + ε), where $\varepsilon =d_{center}^{2}A/I$ and *A* is the cross‐sectional area of the NW. The strength of the NW is thus determined by,^32^

(7) $$\sigma =4F_{center}L\;g\left(\alpha \right)/\pi d^{3},$$

where $g(\alpha )=4/\sqrt{\alpha }\tanh(\sqrt{\alpha }/4)+(2+\cosh(\sqrt{\alpha }/4)-6\sinh(\sqrt{\alpha }/2)\sqrt{\alpha })/\alpha \cosh^{2}{\left(\sqrt{\alpha }/4\right)}^{1/2}.$ A previous study showed that the modulus derived from the bending‐only model could overestimate the strength of the NW by an order of magnitude.^31^ As a result, the bending‐tension model has now been widely accepted for characterizing the modulus of various NWs, including Au,^44^, ^45^ Ag,^46^ Ge,^32^ Si,^31^, ^47^ WO_3–x_,^48^ and ZnO.^49^

#### 2.1.2 Contact Resonance (CR) ‐ AFM Measurement

**Figure 3:**
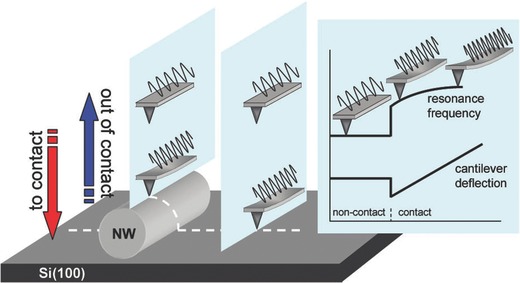
The CR‐AFM method was used to test a NW. The AFM probe contacts directly with the substrate, as well as over the NW. The resonance frequency and deflection of the AFM cantilever were both recorded as the probe was gradually brought in and out of contact with the sample being tested. Reproduced with permission.^56^ Copyright 2016, ACS.

The CR‐AFM (also termed modulated nanoindentation) technique was initially developed for measuring the surface modulus of a material,^50^, ^51^, ^52^ and was later used for measuring the radial elasticity of multi‐wall carbon nanotubes by Palaci et al. in 2005.^53^ In this method, an AFM probe, with oscillation that is driven by modulated signals, first scans the substrate, followed by the NW being tested, as shown in **Figure** **3**. The resonance frequency and deflection of the AFM cantilever are both recorded as the probe is gradually brought in and out of contact with the sample being tested. The resonance frequency changes as a function of the material being probed (substrate or NW), the contact geometry, and the applied force. The normal force of contact can be obtained through measuring the deflection of the cantilever probe. Using the Hertzian contact theory, the contact stiffness between the AFM tip and both the substrate and cylindrical NW can be determined. The radial modulus of the NW is thus derived as,

(8) $$E_{\mathrm{NW}}={\left(\gamma^{-1/2}{\left(k_{T-NW}^{*}/k_{T-S}^{*}\right)}^{3/2}\left(1/E_{S}^{*}+1/E_{T}^{*}\right)+\;1/E_{T}^{*}\right)}^{-1}.$$

where *E* and ν are the Young's modulus and Poisson's ratio of the materials indicated by their subscript indices: *T* for the tip and S for the substrate. The geometrical factor, γ, is dependent on the radii of the tip and the NW, $k_{T-NW}^{*}\;$ and $\;k_{T-S}^{*}$ are the stiffness values between the tip and the NW, and between the tip and the substrate, respectively. This non‐destructive method has been used to measure the elastic properties of various NWs, such as ZnO,^54^, ^55^ oxidized Si^56^ and Si.^57^ The measured values of elastic modulus are in good agreement with those measured using the direct AFM method.

Overall, AFM‐based tests represent a general technique for measuring the mechanical properties of NWs, especially for the accurate measurement of the modulus of NWs. To achieve a reliable measurement, two primary issues must be carefully considered for practical testing. First, if a NW specimen is clamped on a substrate via deposition, the deposited and substrate materials should be stiffer than the NW. Soft deposition or substrate materials can lead to unreliable clamping, and thus a significant overestimation of elastic modulus.^45^, ^47^, ^58^, ^59^ Second, residual stresses can be easily induced in doubly clamped NWs during clamping or subsequent depositing. Under this circumstance, more complex models should be used for calculating modulus from the *F‐δ* curve.^45^, ^58^, ^59^ In addition, it is worth noting that for AFM‐based testing, the measured modulus is always inversely proportional to the fourth power of the NW diameter. Consequently, the derived modulus will be particularly sensitive to the accuracy of the measured diameter. Smaller sample diameters are likely to see the larger scatter in the measured modulus values.

### 2.2 Nanoindentation Based Measurement

**Figure 4:**
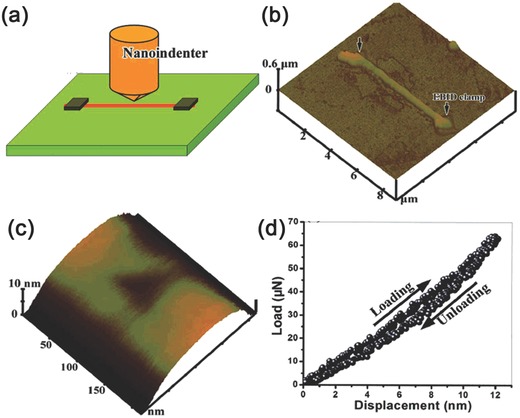
a) The schematic of nanoindentation set‐up. b) 3D AFM image of a clamped Mg_2_B_2_O_5_ NW. c) Representative 3D AFM image of the indentation impression made on the Mg_2_B_2_O_5_ NW. d) Representative load‐displacement curve. Reproduced with permission.^61^ Copyright 2008, ACS.

Nanoindentation is a popular method for characterizing the mechanical properties of materials on a small scale. The testing is convenient and only requires simple sample preparation. In a typical nanoindentation test, a NW is laid on a flat, smooth substrate. Typically the adhesion between a NW and the substrate is sufficiently strong to hold the NW during testing. Nevertheless, improved fastening can be achieved, for example, by electron‐beam deposition of a suitable material at the contact area, usually at the two ends of the NW. A sharp indenter can be used for indentation, and the subsequent indentation impression can be observed by in situ AFM imaging, as shown in **Figure** **4**. The elastic modulus of the NWs is calculated from the recorded load‐displacement curve using the Oliver–Pharr data analysis method.^60^

During indentation testing, the nanoscale probe perpendicularly penetrates into the NW surface. This can avoid the occurrence of slip‐friction sometimes observed during contact between an AFM tip and NW surface in AFM based tests. As a result, nanoindentation has been widely used for directly measuring the mechanical properties of a broad range of NWs, including Ag,^62^ Au,^63^ In,^64^ W,^65^, ^66^ Si,^31^ GaN,^67^ ZnO,^67^ Al_4_B_2_O_9_,^68^ Al_18_B_4_O_33_,^68^ Mg_2_B_2_O_5_.^61^ In addition, nanoindentation with a flat indenter can be used for buckling testing of vertically aligned NWs grown on substrates.^69^, ^70^, ^71^ In the past decade, nanoindentation was successfully integrated with other facilities such as Raman spectroscope,^72^ SEM^73^, ^74^, ^75^ and TEM.^76^ These modern advanced integrated facilities provide powerful tools for developing an improved understanding of the mechanical properties of nanomaterials and nanostructures.

**Figure 5:**
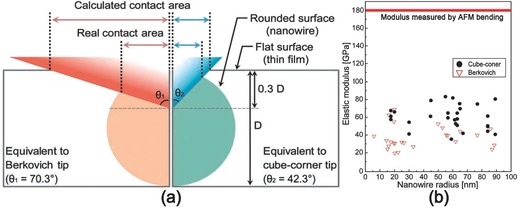
The rounded surface effect on the contact area: a) Schematic illustration of contacts made in the Berkovich (left‐hand side) and cube‐corner indentation (right‐hand side) on both rounded and flat surfaces. b) Elastic modulus obtained from nanoindentation with the two tips. The red line in (b) shows the average value obtained by AFM bending. Reproduced with permission.^31^

It should also be noted that nanoindentation tests suffer from the lack of a standard calibration method for removing uncertainty related to the substrate effects introduced by both the NW surface and the supportive substrate. Kim et al. undertook a comparative test of the elastic modulus of Si NWs using both AFM bending and nanoindentation. The study found that the modulus values obtained by nanoindentation, 37.9 ± 13.3 GPa for Berkovich tip and 59.5 ± 13.3 GPa for cube‐corner tip, were significantly lower than the values of 141.9 ± 33.3 GPa obtained from AFM bending.^31^ The results, as shown in **Figure** **5**, suggested that the rounded surface effect might lead to a significant underestimation in the measurement of elastic modulus. Other comparative tests and corresponding finite element modelling also showed that the modulus values obtained from the Oliver‐Pharr method without consideration of substrate effects might be significantly underestimated. The studies demonstrated that a standard testing protocol is badly needed for NWs.^77^, ^78^, ^79^ It has been shown that the confinement around the indentation volume can dramatically change the underlying deformation mechanism.^80^, ^81^, ^82^ Due to the significant difference in confinement between the bulk materials and NWs during the indentation tests, it is reasonable to speculate that a poorly confined NWs is a potential source for under predicting modulus. Furthermore, despite the simplicity of the experimental technique, indentation with sharp indenters induces complex loading conditions as well as a severe plastic/elastic gradient, which in turn makes it very challenging for modeling and quantitative analysis.

### 2.3 In Situ Electron Microscope (EM) Tests

By integrating an AFM, a nanoindenter/nanomanipulator, or MEMS device into an electron microscope,^76^, ^83^, ^84^ either SEM or TEM, the testing process can be visually monitored. Such systems are termed in situ EM testing. In situ EM techniques allow for observation of the real‐time atomic‐scale deformation of NWs under load, and thus provide a powerful tool for understanding the mechanical properties of NWs.

#### 2.3.1 In Situ SEM Tests

**Figure 6:**
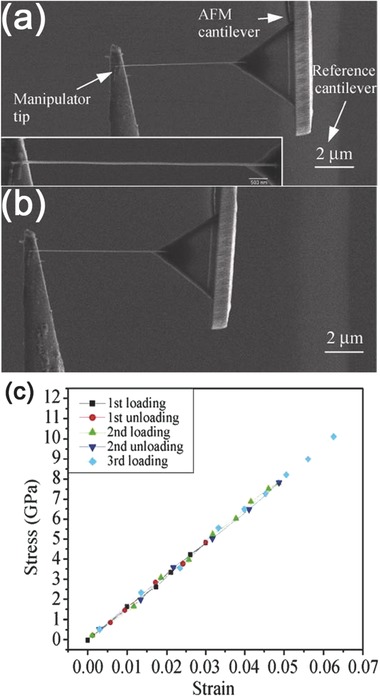
a,b) SEM images a ZnO NW attached between a flexible AFM cantilever and a nanomanipulator during tensile testing. Inset of (a) shows the high‐resolution SEM image of the NW. c) The stress‐strain response of the ZnO NW under repeated loading and unloading. Reproduced with permission.^97^ Copyright 2010, Springer.

Nanoscale tensile testing of nanotubes was performed using AFM manipulation in a SEM by Yu et al.^85^ This technique has now become a widely used method for measuring a variety of NWs including Si,^75^, ^86^, ^87^, ^88^ Ge,^89^ Au,^90^ Ag,^91^, ^92^ Cu,^93^, ^94^ Co,^95^ Ni,^96^ ZnO,^97^, ^98^ GaN.^99^ During testing, the two ends of a NW are clamped onto two respective AFM cantilevers (one ‘stiff’, and the other ‘flexible’) via EB‐induced deposition. The NW is loaded under tension by precise movement of the ‘stiff’ AFM cantilever (which can also be replaced by a nanomanipulator), and the ‘flexible’ AFM cantilever functions as a load sensor by measuring its defection. **Figure** **6** shows a typical SEM images of tensile testing and the corresponding recorded stress‐strain curves.^97^

**Figure 7:**
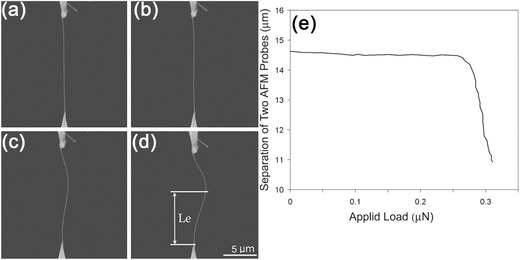
SEM images of a boron NW attached between two AFM cantilevers during a buckling test. The NW (a) is straight, in stable equilibrium, (b,c) starts to bend but remains in neutral equilibrium, and (d) loses its stable equilibrium and buckles with disturbance. e) Separation of two AFM probes as a function of applied load. Reproduced with permission.^100^

The experimental configuration for tensile testing can also be applied to buckling testing of a NW. This is achieved by moving the nanomanipulator toward the other AFM cantilever in order to apply a compressive load to the NW. During compression, bucking occurs, and the critical buckling load is similarly obtained from the deflection of the AFM cantilever. **Figure** **7** shows typical SEM images of a NW in a buckling test as well as the corresponding stress‐strain curve. The elastic modulus of the NW is obtained from Euler's buckling model,^100^

(9) $$E=P_{\mathrm{cr}}L_{e}^{2}/\pi I,$$

where *P*_cr_ is the critical buckling load, *L*_e_ is the effective length (*L*_e_ = 0.5*L* for a fixed‐fixed boundary condition in Figure 7, where *L* is the actual length of the NW). The in situ SEM buckling test was used for measuring the mechanical properties of ZnO,^97^ B,^100^ and Si.^75^ To guarantee the accuracy of these two experiments, it is critical to ensure that the specimens experience uniaxial loading conditions; this can be significant challenge.

**Figure 8:**
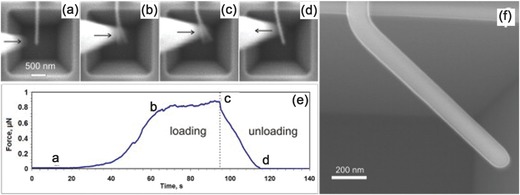
AFM Bending test based on in situ SEM technique. Reproduced with permission.^92^ Copyright 2014, Elsevier. a–d) Selected video frames during bending of an Ag NW and (e) its corresponding force time history. f) A high resolution SEM image shows the bent NW after testing.

Similarly, bending of a NW can be performed using the in situ SEM technique by pushing the free end of a NW, as shown in **Figure** **8**. Based on the force‐displacement relation recorded from the tip deflection, the elastic modulus, yield strain, and yield strength can be derived using Equation (1), (2), and (3) respectively. Compared to the direct AFM bending test, this method can significantly reduce the error originating from relative lateral movement between the AFM tip and NW, as in situ SEM can observe such a slip. With the rapid development of MEMS technology in recent years, MEMS based manipulation devices have been successfully integrated into a SEM for characterizing the mechanical properties of NWs. The function of the MEMS devices is the same as the AFM based set‐up, but more specific to the testing purpose. Certainly, most MEMS devices used inside a SEM can also be integrated into a TEM.^14^ Therefore, MEMS based measurements will be reviewed in conjunction with in situ TEM measurements in the next section in order to avoid repetition.

#### 2.3.2 In Situ TEM Tests

**Figure 9:**
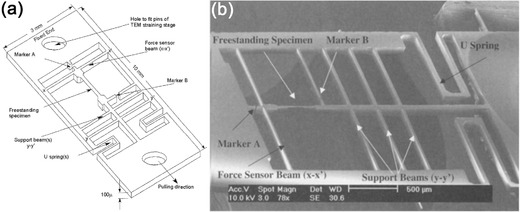
a) Schematic illustration and b) SEM image of the MEMS device for in situ TEM tensile test. Reproduced with permission.^13^ Copyright 2002, Springer.

TEM can examine the microstructure of materials at an atomic scale. In recent years both MEMS and AFM based nanomanipulation systems were successfully integrated into a TEM for in situ mechanical testing of NWs. In situ TEM tests provide an ideal tool for studying the actual deformation mechanisms involving the dislocation activities of NWs under tension/compression. In fact, in situ TEM techniques have now become the most widely used method for characterizing a range of NWs, such as Si,^101^, ^102^ GaAs^103^, ^104^, ^105^, ^106^ ZnO,^107^, ^108^ VO_2_.^109^ GaN,^99^, ^110^, ^111^ ZnTe,^112^ Ag,^113^, ^114^, ^115^, ^116^, ^117^ Ni^118^, ^119^ Cu,^120^, ^121^, ^122^ and metallic glasses.^123^, ^124^ **Figure** **9** shows an SEM image of the first MEMS device successfully integrated into a TEM for in situ nanoscale mechanical testing by Saif et al.^13^ The freestanding specimen being tested was fabricated together with the MEMS structure, and is suspended at the center of the MEMS device. During loading, the gap between the sensing beams change in proportion to their stiffness, while the stiffness of the sensing beams is derived from the cantilever beam theory.^13^ In situ TEM mechanical testing techniques have been well developed in the last several years, and a selection of in situ TEM testing systems are now commercially available.^14^ Typical examples include the NanoFactory TEM‐STM (scanning tunneling microscopy)/AFM holder,^112^, ^125^ Hysitron Picoindenter^126^, ^127^ and various MEMS‐based nanoscale material testing systems.^13^, ^107^, ^108^

**Figure 10:**
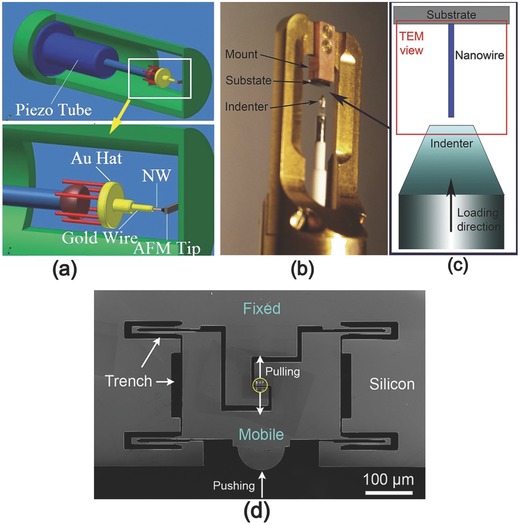
a) A schematic illustration of an in situ AFM–TEM holder used for mechanical measurements. Reproduced with permission.^112^ Copyright 2012, RSC. b) The Hysitron PI 95 TEM PicoIndenter and c) the corresponding schematic of a compression test. Reproduced with permission.^126^ Copyright 2016, Elsevier. d) A PTP device. The black areas are empty space and the dark gray areas are silicon. When the device is pushed on the semicircle end of the mobile part (arrow at bottom), the gap indicated by a yellow circle expands and converts compressive force into tensile force. Reproduced with permission.^109^, ^126^ Copyright 2011, ACS and 2016 Elsevier.

**Figure** **10**a shows an AFM Nanofactory holder that can be used to measure the elastic modulus of NWs.^112^ During testing, a gold NW is adhered to a piezo‐driven movable manipulator attached to the holder, which can precisely move the NW towards the AFM tip in order to make contact between the tip and an the NW. Bending is induced in the NW by pushing of the tip. The elastic modulus of the NW is calculated from the obtained *F* − δ curve. Figure 10b shows an optical image of a Hysitron PI 95 TEM PicoIndenter^©^ and Figure 10c illustrates the working mechanism of the instrument for compression testing. During testing, a NW attached to a substrate has its axis orientated perpendicular to the surface of the piezo‐driven diamond flat punch. By moving the punch toward the NW, a uniaxial compressive load is applied to the NW. In response to a gradually increased compressive load, buckling of the NW will occur. The applied force and displacement of the punch are recorded simultaneously during the process. The elastic modulus can be calculated from the critical buckling load using Equation (9). Figure 10d shows an SEM image of the Hysitron PicoIndenter with a push‐to‐pull (PTP) device, which can also be used in a TEM for studying the tensile strength of NWs. The NW is placed into the gap with each end adhered to the mobile and fixed segment of the PTP respectively, as indicated in Figure 10d by a yellow circle. When the mobile segment is pushed toward the fixed segment, the gap extends, converting the compressive force into tensile loading of the NW bridging the gap.

**Figure 11:**
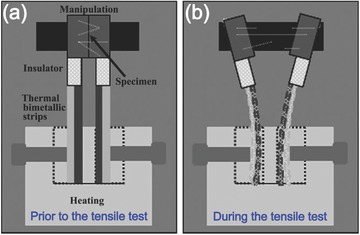
Schematic illustration of thermally driven tensile mechanism for in situ TEM testing of NWs. Reproduced with permission.^128^

The integration of a manipulator into a TEM typically affects the tilting functionality of the TEM, making specimen observation inconvenient. To solve this problem, special set‐ups that do not sacrifice the tilting capability of in situ TEM observations were developed. Based on a thermal bimetallic technique, Han et al. developed a novel controllable tensile testing device in a TEM, which deforms a NW sample at very slow strain rates.^128^ As shown in **Figure** **11**, the device has two thermally actuated bimetallic strips with different thermal expansion coefficients and a NW sample is attached to the edges of a trench between the bimetallic strips. The bimetallic strips slowly bend in opposite directions when heated on a TEM heating stage with double tilting capability. This pulls the specimen at a strain rate that can be controlled by adjusting the temperature of the heating stage. The approach is useful for understanding material deformation mechanisms at atomic scale, but cannot provide quantitative stress analysis.

#### 2.3.3 The Challenges of EM Tests

**Figure 12:**
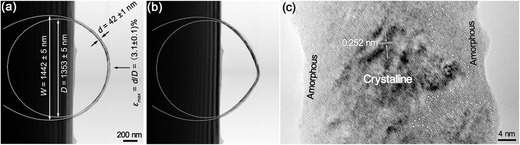
a) TEM image of a looped NW, b) the NW loop is distorted after exposure to TEM electron beam for 1 hour, c) high resolution TEM image showing amorphous phase being induced. Reproduced with permission.^145^

**Figure 13:**
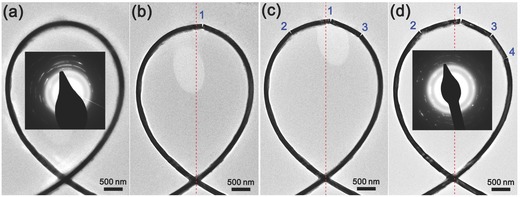
TEM images of (a) A NW loop without fracture just at the beginning of imaging and b–d) the same NW loop exposed to electron beam during TEM imaging for different times, with fracture being generated on the NW. Reproduced with permission.^146^ Copyright 2013, IOPscience.

In situ EM testing enables direct observation of the deformation of tensile/compressive NWs at atomic scale. This thus provides a powerful approach for studying the deformation mechanisms, enabling the discoveries of novel mechanical behaviors of NWs such as anelasticity,^103^, ^129^, ^130^ recoverability,^115^, ^131^ super plasticity^120^, ^132^ and super elasticity.^133^ However, significant challenges still remain with respect to sample preparation and real‐time characterization of the microstructural changes in strained NWs.^134^ Also, as the mechanical test is performed in an environment containing a high‐energy electron beam, attention must be paid on how electron beam irradiation (EBI) would affect the mechanical properties of NWs.^83^, ^135^ Thermal effect and knock‐on effect from energetic electrons in TEM or SEM may contribute to NW specimens damage.^136^ Thermal effect induced by energetic electrons is difficult to quantify, however recent studies have revealed that the temperature increase of a NW sample during a normal TEM imaging process is far below 100 °C.^99^, ^102^, ^137^, ^138^ Therefore thermal effect should insignificantly alter the properties of NWs. Knock‐on effects can be evaluated by considering the threshold displacement energy (TDE), that is the minimum amount of kinetic energy transferred to a lattice atom resulting in the formation of a stable Frenkel pair.^136^ However, sub‐threshold irradiation, which can significantly affect defect migration or induce local chemical reactions in NW samples,^136^, ^139^, ^140^, ^141^ has rarely been considered with regards to strained NWs.^138^, ^142^ In addition, it should be noted that the existing TDE value for a specific material is measured in a stress‐free state, which might not hold for specimens under strain. In fact, recent theoretical analyses and simulations have demonstrated that the TDEs of strained nanostructures would decrease with an increase in strain,^143^ suggesting that it is easier to damage a strained NW by EBI.^144^ This was demonstrated by a recent study shown in **Figure** **12**, where amorphization occurred in a looped single crystal SiC NW under a maximum strain of 3.1% during TEM characterization.^145^ Beside EBI‐induced amorphization, fracture of the strained NW could also be induced by EBI. As shown in **Figure** **13**, fractures were generated in an Al_2_O_3_ NW loop when it was examined during TEM imaging.^146^ Previous studies have also shown that EBI may increase the plasticity of NaCl,^142^ SiO_2_ 138 and Si NWs,^147^ weaken the strength of Al_2_O_3_ NWs,^146^ stiffen zinc tin oxide NWs,^148^ and significantly decrease the fatigue life of ZnO NWs.^149^ Also, EBI during SEM imaging may generate pits in the shell structure of carbon nanotubes.^150^ In order to clarify the effects of high energy electrons, ‘beam off’ tests are usually necessary for in situ EM mechanical tests. Usually, materials with metallic bonds are less likely to be affected by an EBI under normal testing conditions. However, EBIs can significantly affect the mechanical properties of materials with ionic or covalent bonds.

### 2.4 Optical Microscope (OM) Based Tests

**Figure 14:**
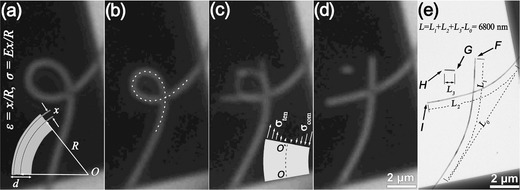
Optical images extracted from the video record of a loop test, showing (a) under loop bending, (b) just prior to wire breaking, (c) immediately after breaking and (d) the final status of the tested NW. e) TEM image of the broken NW. Reproduced with permission.^145^

Two OM‐based tests have successfully been developed for the mechanical measurement of NWs. The first is an OM‐based resonance test, used for characterizing the elastic modulus of NWs. the OM‐based resonance test was used for characterizing the elastic modulus of NWs in an accurate manner. In this method, the vibration spectra of a NW cantilever were obtained using optical interferometry, and the elastic modulus of NWs can thus be derived from the Euler–Bernoulli beam theory.^151^, ^152^, ^153^, ^154^ Detail information on this method will be introduced in Section 2.5. The second method is an OM‐based loop test for measuring the fracture stress and strain of brittle NWs.^145^, ^146^ A NW is first placed on the suspending film of a TEM grid with an atomically smooth surface, and then manipulated into a loop, as shown in **Figure** **14**. Pulling on one end of the NW decreases the size of the loop until failure occurs. The pulling process is monitored under an optic microscope. The radius of the top half of the NW loop prior to fracture, *R*_min_, also named as the Sinclair circle, is defined as,^146^, ^155^

(10) $$R_{min}=0.469W_{min}=L/8,$$

where *W*_min_ is the width of the loop prior to breaking, and *L* is the overlapped length of the NW after breaking, as defined in Figure 14e. *R*_min_ can be determined if *W*_min_ or *L* is known. For a NW of *d* in diameter, the maximum tensile stress σ_max_ and strain ε_max_, is experienced at the outer edge of the bending apex of the NW, which are given by,^156^

(11) $$\varepsilon_{\max}=x/R_{\min},\;\sigma_{\max}=E\varepsilon_{\max}.$$

where *E* is the elastic modulus of the NW.

The OM‐based tests are simple and reliable, and can be performed under an air atmosphere. This could be an effective method for exploring the effects of testing environments such as humidity and temperature on the mechanical behaviors of NWs. Without the effects from EBI, the OM‐based loop test can be used to study the mechanical behaviors of NWs such as brittle‐to‐ductile and crystalline‐to‐amorphous transitions and anelasticity. By combining with in situ EM characterization, such a loop test can be a convenient tool for understanding the microstructural changes induced by EBI in the strained nanomaterials. Nonetheless, it should be noted that due to the resolution limitation of optical microscopy it would be extremely challenging to study NWs with diameters smaller than 30 nm.^145^

### 2.5 Resonance Tests

Resonance testing was often used to calibrate the spring constant, and hence the bending modulus of AFM cantilevers.^157^ The use of resonance for measuring the elastic modulus of one‐dimensional nanostructures was first carried out in TEM by Treacy et al.^158^ and was later improved by integrating the electrostatic excitation into the EM based testing process.^159^ During testing, the resonant vibration of a NW cantilever was induced by matching the alternating electric field with its natural frequency. The elastic modulus of the NW material can be derived from the Euler–Bernoulli beam theory, defined as,^160^, ^161^

(12) $$E=\frac{2\pi^{2}L^{4}f_{i}^{2}\rho A}{\alpha_{i}^{4}I},\;\;i=1,2,3\ldots \ldots$$

where *f_i_* are the eignfrequencies (where *i* is the mode number), *L* the resonating length, *I* the second moment of area, *A* the area of the cross‐section, ρ the density of the NW, and α_*i*_ (= 1.875, 4.694, 7.855, … for *i* = 1, 2, 3, 4, …) are the constants satisfying the transcendental equation of cosα_*i*_coshα_*i*_ + 1 = 0.

Unlike in situ EM techniques, scanning laser Doppler vibrometry (SLDV) is an alternative tool that measures the vibration of a micro‐ and nano‐ structure with 1 picometer resolution in the detection of vibration amplitude.^140^, ^162^, ^163^ SLDV uses the Doppler shift of the reflected laser beam from a vibrating object to measure that object's vibrational velocity. The eigenmodes and corresponding quality factors of a vibrational object can then be precisely determined. SLDV techniques have recently been used to measure the vibration of nanostructures with diameters smaller than 50 nm.^3^, ^151^, ^152^, ^153^, ^154^ The high resolution of SLDV techniques allows for a simple experimental setup. In a SLDV test that is capable of detecting thermally driven vibration, the self‐adhesion between a NW and a substrate is usually sufficiently strong to hold the NW in place.^160^, ^164^ However, if an external excitation source is required, than such clamping may not be sufficient.^165^

**Figure 15:**
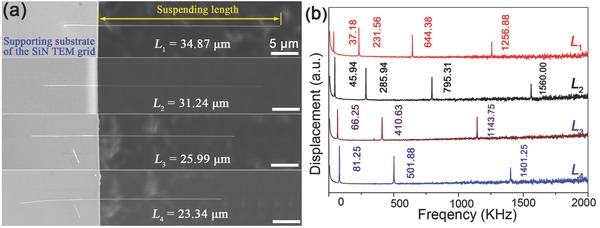
a) SEM images of four Al_2_O_3_ NW cantilevers of different resonating lengths and b) their corresponding frequency spectra. Reproduced with permission.^164^ Copyright 2016, IOPscience.

By examining the ratios of resonant frequencies of a NW cantilever, the effect of clamping or geometric uniformity on testing accuracy can be precisely determined if SLDV is used. This is because SLDV‐based testing enables exact determination of the various vibration modes of a NW cantilever. If a NW cantilever is well clamped and its dimensional inaccuracy can be neglected, the ratios of the measured resonant frequencies should be constant, defined as,^160^

(13) $$f_{1}:f_{2}:f_{3}:f_{4}\cdots =1:6.265:17.554:34.402\cdots \;$$

where *f_i_* (*i* = 1, 2, 3, 4…) is the resonant frequency of i^th^ vibration mode of the NW cantilever. **Figure** **15** shows a SEM images of Al_2_O_3_ NW cantilevers of different resonating lengths and the corresponding frequency spectra.^164^ The ratios of the measured resonant frequencies for the 4 resonating lengths are1: 6.23: 17.33: 33.81,1: 6.22: 17.31: 33.95,1: 6.20: 17.26: 34.08 and1: 6.12: 17.25, respectively. The differences between the measured ratios and the theoretical values defined in Equation (13) are smaller than 2%. This indicates that the NW cantilevers were well‐clamped and should have highly uniform cross‐sections.

Owing to its simplicity and efficiency in operation, the resonant vibration testing was widely used to determine the elastic modulus of NWs including Si,^154^, ^166^ Ge,^167^ Co,^168^ W,^161^, ^169^ SiC,^170^ GaN,^99^, ^171^ Ag_2_Ga,^154^ BN,^172^ ZnO,^149^, ^165^, ^173^, ^174^ WO_3_,^175^ In_2_O_3_,^168^ Sb_2_O_3_,^176^ Al_2_O_3_,^164^ amorphous SiO_2_,^177^ metallic glasses.^153^ In particular, the use of SLDV not only significantly increases the detection resolution of a NW specimen's vibrational spectra, but also eliminates the issues relating to clamping and dimensional non‐uniformity.^154^, ^161^, ^164^ Moreover, the resonance‐based testing can be performed without contact of a NW, thus not necessitating the consideration of temperature effects on force and displacement measurements. So the resonance‐based test may be used for future studies on the temperature‐dependent elasticity of NWs.

## 3 Mechanical Characterization of NWs

The mechanical characterization demonstrated that most of the NWs being tested could experience extremely high stress closing to their respective intrinsic strengths without exhibiting inelastic relaxation such as plasticity or fracture, and some NWs exhibited significant great anelasticity and superplasticity. This offers new opportunities for developing new materials for electronic, optical, magnetic, photonic, and catalytic applications, through controlling the elastic strain inside NWs.^178^ This echoes the Richard Feynman's statement, “there's plenty of room at the bottom.”^179^

### 3.1 Elasticity of NWs

The measured elastic modulus of a bulk material reflects the intrinsic elasticity of the material. The measured elastic modulus of a NW may be quite different from its bulk counterpart, and is often dependent on the size, structure and defect within the NW, as well as the mechanical testing strategy and environment.

#### 3.1.1 Effect of Surface on Elastic Modulus

Atoms at the surface or interface of a material system experience a different local environment to those contained within the volume. Therefore, the energy associated with the surface atoms is generally different from that of the bulk atoms. As surface atoms are contained within a layer typically only several nanometers thick, their effect cannot be detected in bulk materials, and are thus neglected in the classical theory of elasticity. However, for a NW with a high surface‐to‐volume ratio, such an effect can no longer be neglected.^180^

Based on continuum mechanics, *nanostructure*  =  *bulk*  +  *surface*, the surface effect on the elasticity of a NW can be described by a core‐shell model,^181^ where the core and shell represent the bulk material and the several nanometer thick surface layer respectively. From the thermodynamic theory,^182^ the total surface stress, τ, associated with the reversible work required to elastically stretch a pre‐existing surface, can be written as,^182^

(14) $$\tau =\tau_{0}+E_{s}\epsilon ,$$

where τ_0_ is a strain‐independent parameter referred to residual surface stress, ε is dimensionless strain and *E*_s_ is surface modulus, and *E*_s_ε is often referred to surface elasticity. The surface elasticity of a material has a different elastic property from that of its bulk material. Surface relaxation and reconstruction are the two fundamental mechanisms responsible for the different property of a surface layer. Both τ_0_ and *E*_s_ε can be either positive or negative, and so τ can either stiffen or soften the corresponding micro/nanostructure. For example, τ is typically tensile for fcc metal NWs, suggesting that the surface could reduce its energy by contracting,^183^, ^184^ however, τ is generally compressive for semiconductor NWs, leading to surface expansion at equilibrium.^107^, ^185^, ^186^

The first core‐shell model used to describing the surface effect on the elastic modulus of a NW was established by Miller and Shenoy.^187^ In this model, the NW consists of a core, with elastic modulus *E*_0_, and a surface, of zero thickness and surface elastic modulus *E_s_*, ideally adhered to the core. The elastic modulus of the NW *E* is given by,^187^

(15) $$E=E_{0}+4E_{s}/D,\;\mathrm{for}\;\;\;\mathrm{tension};$$

(16) $$E=E_{0}+8E_{s}/D,\;\mathrm{for}\;\;\;\mathrm{bending}.$$

The assumption of “zero” thickness might be unrealistic, so an improved core‐shell structure is composed of a core and a surface shell layer with constant thickness, *r_s_*. The effective elastic modulus of a NW under tension and bending can be calculated as,^97^, ^173^

(17) $$E=E_{0}\left[1+4\left(E_{s}/E_{0}-1\right)\left(r_{s}/D-r_{s}^{2}/D^{2}\right)\right],\;\mathrm{for}\;\;\;\mathrm{tension};$$

(18) $$\begin{array}{l} E=E_{0}[1+8\left(E_{s}/E_{0}-1\right)\\ \left(r_{s}/D-3r_{s}^{2}/D^{2}+4r_{s}^{3}/D^{3}-2r_{s}^{4}/D^{4}\right)],\;\mathrm{for}\;\mathrm{bending}. \end{array}$$

The core‐shell model defines a discontinuity in the elastic modulus value at the interface between the core and shell. As a result, a modified core‐shell model was proposed, introducing the concept of inhomogeneous surface elasticity under the continuum theory framework. The elastic modulus of a circular NW is thus expressed as,^188^

(19) $$\begin{array}{l} E=E_{o}\{{\left(1-2r_{s}/D\right)}^{2}+4r_{s}/\alpha D\left[e^{\alpha }-\left(1-2r_{s}/D\right)\right]\\ \;\;\;\;\;\;\;-8{\left(r_{s}/\alpha D\right)}^{2}\left(e^{\alpha }-1\right)\},\;\mathrm{for}\;\;\;\mathrm{tension}; \end{array}$$

(20) $$\begin{array}{l} E=E_{o}\{{\left(1-2r_{s}/D\right)}^{4}\;+8r_{s}/\alpha D\left[e^{\alpha }-{\left(1-2r_{s}/D\right)}^{3}\right]\\ \;\;\;\;-48{\left(r_{s}/\alpha D\right)}^{2}\left[e^{\alpha }-{\left(1-2r_{s}/D\right)}^{2}\right]\\ \;\;\;\;+192{\left(r_{s}/\alpha D\right)}^{3}\left[e^{\alpha }-\left(1-2r_{s}/D\right)\right]\},\;\;\;\mathrm{for}\;\mathrm{bending}. \end{array}$$

where α is a dimensionless constant describing the degree of inhomogeneous elasticity in the transition from the bulk region to the external surface layer. The sign of α, positive or negative, describes stiffening or softening of the surface layer respectively. Although Equations (15)–(20) could predict the tendency of surface effect on the elasticity of NWs, the parameters including surface modulus, shell thickness, and degree of inhomogeneous elasticity must be determined by theoretical calculation, simulation, or experimental measurement.

Theoretical calculation and simulation methods, such as molecular dynamics (MD) simulation based on embedded‐atom‐method interatomic potentials and Ab initio calculation, are used to understand the surface effect on the elastic modulus of NWs. MD simulation can predict the overall response by considering the surface as a separate layer of predetermined thickness from the core material.^187^ Size‐dependent surface modulus thus plays a significant role in determining the elasticity of NWs. In the Ab initio model, the relation between the redistribution of surface electron density and the elasticity of a NW is established, and the orientation‐dependent electron redistribution can increase or decrease surface stiffness.^189^ Molecular statics simulation also shows that the nonlinear elastic response of a NW core can affect the elastic modulus of some NWs, including Cu,^190^ Ag,^191^ ZnO^192^ and SiC.^193^ This is attributed to tensile surface stresses that can exist in a NW in its equilibrium state, producing axial compressive strain at the core, leading to an inherent nonlinear elastic response.

Although theoretical calculations and numerical simulations have provided in‐depth understanding of the surface effect on NW elasticity, such predictions are heavily dependent on the type of model used and interatomic potential assumptions. This may generate contradictory results. For example, MD simulation using Buckingham‐type interatomic potential showed that the elastic modulus of [0001]‐oriented ZnO NWs increased from 140 to 340 GPa when the NW diameter shrank from 3 to 1 nm^192^ and a large‐scale MD simulation using the same potentials showed that the elastic modulus of ZnO NWs increased from 169 to 194 GPa as the NW size decreased from 20 to 5 nm.^107^ However, the First‐principles calculation based on the modified Lewis potential produced the elastic modulus of ZnO NWs smaller than that of the bulk counterpart and the modulus value decreased from 121.5 to 96.7 GPa when the NW size shrank from 6 to 1.8 nm.^194^

**Figure 16:**
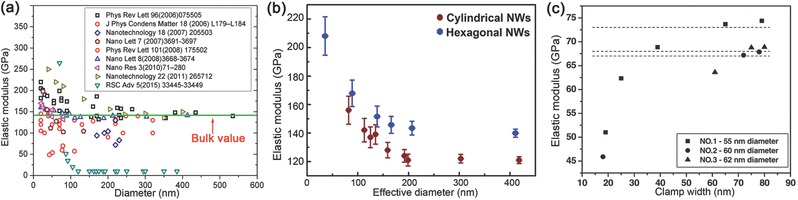
a) The elastic moduli of ZnO NWs obtained using various methods plotted as a function of NW diameter. b) Elastic moduli of the cylindrical and hexagonal ZnO NWs fabricated by different conditions plotted as the NW effective diameter. Reproduced with permission.^201^ Copyright 2015, ACS. c) Effect of clamping on the elastic moduli of ZnO NWs obtained from the resonant vibration test. Reproduced with permission.^165^

A great number of experimental studies have been performed for characterizing the surface effects on the elasticity of NWs. Many of those results indicated that the elastic modulus of ZnO NWs increased with the NW size shrank owing to the enhancement of surface elasticity,^55^, ^107^, ^173^, ^195^ while the elastic modulus of Si NWs decreased with the decreased diameter.^86^, ^196^ Nevertheless, the reported modulus data of ZnO NWs exhibit quite large scattering, as shown in **Figure** **16**a. Also can be seen in Figure 16a, the decreasing rate of modulus with the NW size is relatively great in some studies,^29^, ^54^, ^174^, ^197^ but some appear to be independent on size,^41^, ^49^ and some had significantly lower values than the bulk moduli.^54^, ^198^, ^199^, ^200^ The discrepancy should be mainly attributed to the measuring uncertainties encountered in practical tests, the appropriateness of testing methodologies, as well as the defects and microstructures of NW samples being used. For example, the slight differences in the microstructures induced by the growth conditions or the small uncertainty in the clamping process might lead to significantly different results in the practical tests, as can be seen in Figure 16b and c, respectively.^165^, ^201^ Certainly, exact determination of the surface effects on the elasticity of NWs still remains a significant challenge.

#### 3.1.2 Effect of Defects on Elastic Modulus

Defects in a crystalline material terminate the periodicity of its atomic arrangement and alter the electron distribution within atomic structure, and thus the elastic modulus of the material. In general, point defects can increase average bond length and thus result in reduction in elastic modulus.^202^, ^203^ Recent theoretical calculations and simulations suggested that the elastic moduli of many NWs decreased significantly with the increase in vacancy densities, including SiC,^204^ GaN,^205^ Si,^206^, ^207^ W,^208^, ^209^ Ag,^191^, ^210^ Au^211^ and Cu.^212^ Experimental results^206^, ^207^ also indicated that vacancies might be one of the important reasons for the relatively low modulus values of the NWs. For instance, ZnO NWs of cylindrical cross‐sections exhibited lower elastic modulus than those of hexagonal cross‐sections, as they had higher density of oxygen vacancies.^201^ In addition, it was also suggested that high‐density oxygen vacancies should be partially responsible for the relatively low modulus values of Al_2_O_3_ NWs.^164^

**Figure 17:**
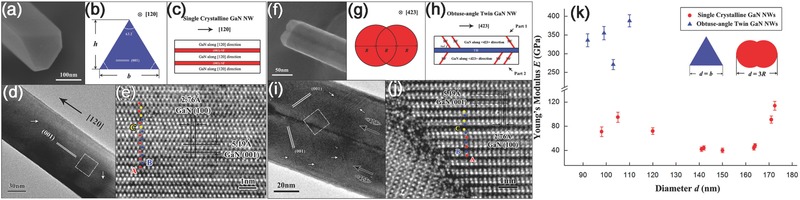
Typical characterization results of single‐crystalline and obtuse‐angle twin (OT) GaN NWs. Reproduced with permission.^99^ a,b) The observed isosceles triangle cross‐section and the corresponding model of single‐crystalline GaN NWs. c) The structural model of the single‐crystalline GaN NWs. d) The TEM image of an individual single‐crystalline GaN NW. White arrows indicate the (001) SFs in the NW. e) The HRTEM image acquired from the rectangular area in (d), showing the change of stacking sequence in the single‐crystalline NWs NW. f,g) The observed dumbbell‐shape cross‐section and the corresponding model of OT NW. h) The structural model of the OT NW. i) The TEM image of an OT GaN NW. White arrows indicate the (001) SFs in the NW. j) The HRTEM image collected from the white area in (i), showing the change of stacking sequence in OT NW. k) Young's modulus ***E*** with diameter ***d*** of GaN NWs.

**Figure 18:**
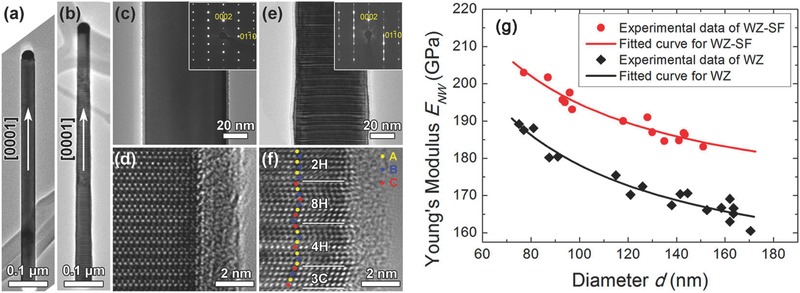
Low‐magnification TEM images of WZ (a) and WZ‐SF (b) GaAs NWs. Diffraction contrast (c) and HRTEM (d) images of WZ GaAs NWs. Diffraction contrast (e) and HRTEM (f) images of WZ‐SF GaAs NWs. g) The effective Young's modulus of WZ (black rhombus) and WZ‐SF (red dot) GaAs NWs as a function of diameter and best fits to the WZ (black solid line) and WZ‐SF (red solid line) data. The insets in (c) and (e) are their corresponding SAED patterns. Reproduced with permission.^105^ Copyright 2016, ACS.

Atomistic simulations revealed that atomic disordering at grain boundaries may significantly alter the interatomic bonding in defected areas, and hence the local elastic moduli within a material.^213^ However, the fundamental mechanism of the defect effects on elasticity remains unclear. For example, the first‐principles calculations of bonds in AlN, InN, GaN, and BeO with wurtzite (WZ) structures suggested that the bonding strength of a ZB‐like region, where stacking faults are often introduced in a WZ structure, is higher than that of a perfect WZ structure.^214^ However, MD simulation suggested that grain boundaries can significantly lower the elastic modulus of GaN NWs, but stacking faults might not significantly change the modulus of SiC NWs.^204^ As a result, the effects of planar defects on the modulus seem to be dependent on the specific microstructures of the NWs being tested. This is also supported by the experimental evidence that planar defects could increase or decrease the modulus values of NWs.^99^, ^105^, ^171^, ^175^ For example, Dai et al. characterized the effects of planar defects on the elastic modulus of GaN NWs, using both in situ TEM vibration and tensile tests (see **Figure** **17**).^99^ For the single‐crystalline GaN NWs of diameters greater than 92 nm, the average elastic modulus is 338 ± 16 GPa, close to the bulk value. The (001) stacking faults parallel to the NW axis could be regarded as the parallel fibers in the Voigt rule of mixtures and the limited quantity of stacking faults did not have an obvious influence on the NW modulus. While for obtuse‐angle twin GaN NWs of diameters ranging from 98 to 171 nm, the elastic modulus is 66 ± 5 GPa. The angle between (001) stacking fault and the NW axis was 54.6°, which decreased the modulus dramatically following the Reuss model.^215^ Chen et al. studied the elasticity of GaAs NWs of two distinct structures, defect‐free single crystalline WZ and WZ containing a high density of SFs, using in situ TEM compression combined with finite element analysis, and found that the presence of a high density of SFs increase the elastic modulus by 13% (see **Figure** **18**).^99^, ^105^ Apparently, a universal rule for interpreting those controversy results is still lacking.

#### 3.1.3 Elastic Strain

Elastic strain is the maximum strain that a material can experience prior to plastic deformation or brittle fracture. Usually a crystalline material cannot sustain elastic shear or tensile strain exceeding 0.2–0.3%, though it is well documented that the theoretic elastic strains of most materials with a perfect crystalline structure are around 10%.^216^ This is attributed to the presence of defects in microstructures. NWs usually possess nearly defect‐free structures, and thus are ideal specimens for experimentally measuring the intrinsic elastic strain of a solid material.^146^ **Table** **1** summarizes the experimental and theoretical elastic strains of typical NWs.

**Table 1:** Summary of elastic strains and strengths of metallic and inorganic NWs. ε_*exp*_ and ε_*theo*_ are the elastic strains, and σ_*exp*_ and σ_*theo*_ are the elastic strengths obtained from experiment and simulation, respectively

NW Materials	ε_*exp*_ (%)	σ_*exp*_ (GPa)	ε_*theo*_ (%)	σ_*theo*_ (GPa)	Microstructure/structure	Testing method
Si	11.5^11^	18.5	≈20^218^	≈ 17	110‐oriented NWs	AFM bending
	16^217^	≈ 20				in situ TEM tensile test
	6.5^86^	12.2	20.7^218^	19	111‐oriented NWs	in situ SEM tensile testing
Ge	17^89^	18	20^219^	14	NWs	in situ SEM bending
	7.5^32^	15			NWs	AFM three‐point bending
ZnO	6.2^108^	9.5	6.5^108^	≈ 13	NWs	in situ TEM tensile testing
	≈7^23^	≈12.1			NWs	in situ SEM bending
ZnS	8.2^220^	0.37			NWs	in situ SEM bending test
GaAs	≈ 7^104^	5.4		5.6^104^	Zinc‐blende structured NWs	in situ TEM compression test
	–	6.2^104^		6.4^104^	Wurtzite structured NWs	in situ TEM compression test
InAs	≈ 10^221^	≈ 5			NWs	in situ tensile test
GaN	2.5^99^	3.1	≈ 30^222^	≈ 35	GaN NWs with planar defects	in situ tensile test
		1.76^111^			single‐crystalline GaN NWs	in situ tensile test
WS_2_	14^223^	16	14.7^223^	40	Nanotubes	in situ SEM tensile test
Silica		26^224^	22^216^	≈ 16	NWs	Tensile test
VO_2_	3.8^109^	5.2^109^			NWs	in situ TEM tensile test
α‐Al_2_O_3_	10.6^146^	48.8	11.5^225^	44.5	NWs	OM‐based loop test
SiC	7^145^	≈ 35	8^204^	≈ 25	NWs with SFs	OM‐based loop test
	10^15^	53.4	≈ 11^204^	28.5^204^	NWs	AFM bending test
Au	≈ 10^43^	≈ 8	≈ 27^216^	25	NWs	AFM bending test
	≈19.6^226^	9.8			polycrystalline NWs	in situ TEM tensile test
Cu	7.2^121^	≈5.8	≈ 8^227^	6.5	single‐crystalline NWs	in situ TEM tensile test
	≈ 5^122^	2.12	≈ 5^228^	3	Twinned nanopillars	in situ TEM tensile test
Ni	≈34.6^133^	≈ 5^229^		≈ 25^229^		in situ SEM tensile test
Nb	4^230^	1.8	–	–	Nb NWs embedded in a NiTi matrix	in situ TEM bending test
Ag	4^116^	4.8	≈ 10^116^	≈ 5.5	Five‐twinned Ag NWs	in situ SEM tensile test
Co	2.14^95^	2.04	≈10^95^	≈ 7.5	NWs	in situ SEM tensile test

**Figure 19:**
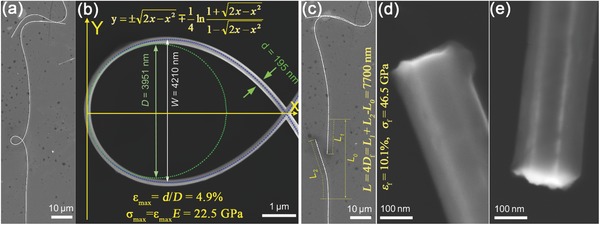
a,b) Low‐ and high‐magnification images of a looped Al_2_O_3_ NW adhered to a Si substrate. The blue dotted curve and the green dotted curve are the theoretical shape and fitted circle at the apex. c–e) Low‐ and high‐magnification images of the two segments from the whisker loop after brittle fracture at a strain of 10.1%. Reproduced with permission.^146^ Copyright 2013, IOPscience.

As can be seen in Table 1, [001]‐oriented Si has a theoretical elastic strain of ≈17%, calculated using the classic Orowan‐Polyanyi method.^216^ MD simulations based on different interatomic potential models showed that the elastic stain of Si NWs was greater than 15%.^185^ Experimental measurements showed different elastic strains for Si NWs, such as 16% from the in situ TEM tensile test,^217^ 12% from the in situ SEM tensile test,^86^ 1.5% from the in situ SEM bending test,^231^ ≈5% from the nanoindentation^31^ and AFM bending tests,^22^, ^31^ and 4.6% and 14.7% from the respective in situ TEM tensile and bending tests.^101^ For SiC NWs, a maximum elastic stain of 10% was observed in an early AFM‐bending test.^15^ This value might be slightly overestimated due to the use of a simple mechanical model. In situ TEM bending tests showed that SiC NWs could experience an elastic strain of ∼2%.^232^ However, the result might be affected by EBI in TEM. Recently, an elastic stain of 4.5% was obtained in an in situ SEM tensile test of SiC NWs,^233^ and ≈7% was measured in the OM‐based loop test,^145^ which are close to the theoretical prediction of 5% using the first‐principles calculation^234^ and 8% obtained from the MD simulation,^204^ respectively. The OM‐based loop test also showed that Al_2_O_3_ NW had an elastic strain of 10.1% (see **Figure** **19**).^146^ The experimentally measurements appear being heavily influenced by methodologies used and environmental conditions under which the tests were performed.

**Figure 20:**
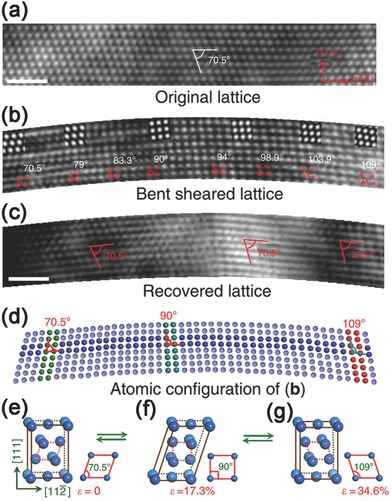
a) HRTEM image of a Ni NW with a bending strain of ≈1.9%. b) The NW lattices with continuous increase in lattice angle α from 71° to ≈109°. The simulated HRTEM images are inserted accordingly. c) HRTEM image captured after the strain released. No square lattice and other type sheared lattices were observed, indicated the 34.6% lattice shear strain was fully recovered. d) Atomic configurations corresponding to (b) to show the ultra‐large continues lattice shear from 0 to 34.6%. e–g) Schematic 3D structures and corresponding side views show shearing and recovery of FCC lattice. The scale bars are for 1 nm. Reproduced with permission.^133^ Copyright 2013, Nature publishing group.

Table 1 also shows that the values of elastic strain of most metallic NWs obtained from either simulation or measurement are below 10%. However, a recent in situ TEM bending test demonstrated that Ni NWs could experience a reversible shear strain up to 34.6%.^133^ In situ TEM examination revealed that in this case the continuous lattice straining in a bent Ni NW started from the face‐centered cubic lattice, then transformed through orthogonal path to a body‐centered tetragonal structure, and finally formed a re‐oriented face‐centered cubic structure, as shown in **Figure** **20**. The reversible strain observed in the bending test is approximately four times of the corresponding values from the theoretical prediction and tensile test,^235^ but the underlying physical explanations are still unclear.^236^

Overall, the measurement of elastic strain of NWs has established a good test‐bed for validating theoretical predictions, which has in turn improved our understanding of the elasticity of nanostructures. As most of the NWs can sustain a shear or tensile strain of exceeding 1%, they can serve as the materials for “elastic strain engineering”, i.e. achieving desired functional properties by controlling elastic strain. This may offer a lot of new opportunities for the applications of NWs.^178^

#### 3.1.4 Factors that Cause Discrepancies in Measurements

The values of elastic modulus and strain of NWs being measured appear to be affected by their geometric shape, size, crystal orientation and surface status. Most NWs have a single‐crystalline structure, but usually possess unavoidable defects of different types and densities, depending on the specific fabrication process. Although defects are believed to be a main source for causing the discrepancies in the reported values of elastic modulus and strain, till now, the defect effects on the measurement have not be determined quantitatively.

Geometric shape can affect the measured values. NWs often have faceted shapes when their diameters are greater than 100 nm, but may have round shapes if their sizes are smaller than 100 nm, in order to minimize their surface energies during growth.^2^, ^237^ Subtle changes in cross‐sectional shape can influence the measured results. For example, Ag NWs usually have a five‐twinned structure with a pentagonal cross‐section if their diameters are greater than 100 nm, which can be precisely determined using SEM. For those smaller than 100 nm, however, it is difficult to distinguish whether their cross‐sections are circular or pentagonal. In fact, Ag NWs can gradually transit their cross‐section from pentagonal to circular when their size gradually shrinks from several hundred nm to tens of nm.^114^ For a circular cross‐section, the area, *A_circ_*, and the area moment of inertia, *I_circ_*, are calculated as,^92^, ^114^

(21) $$A_{circ}=\pi D^{2}/4,\;I_{circ}=\pi D^{4}/64,$$

where *D* is the nominal diameter that can be measured from SEM/TEM images. For a pentagonal cross‐section, the area, *A_pent_*, and the area moment of inertia, *I_pent_*, are expressed as,^92^, ^114^

(22) $$\begin{array}{l} A_{pent}=5D^{2}/8\sin\left(2\pi /5\right),\\ I_{pent}=A_{pent}D^{4}\left[1+3\cot^{2}\left(\pi /5\right)\right]/192\sin^{2}\left(3\pi /10\right), \end{array}$$

Therefore, if we use Equation (22) to calculate the modulus of Ag NWs of diameters below 100 nm, an overestimate will be produced, leading to an error of 18% for tensile testing, 9% for vibration testing, and 41% for bending tests.^92^, ^114^

The surface layer of a NW is another important factor. Oxide layers always exist on the surfaces of semiconductor and metallic NWs. The surface of a NW may also be contaminated during the fabrication and measurement process. Such a surface layer usually has a thickness of several nm, but it can significantly affect the modulus values being measured, such as the cases of measuring GaAs,^106^, ^238^ Si,^22^, ^239^, ^240^, ^241^ W,^161^ Ag^25^ and Al_2_O_3_ NWs.^164^ Although NWs with a surface layer can be modelled as a core‐shell structure, those models still assume that the surface layer is homogeneous and has a constant modulus. In fact, a recent study showed that the elastic modulus of the surface layer may increase three‐folds if the layer thickness reduces from 5 to 2 nm.^106^ It thus remains a great challenge to accurately characterize the elastic modulus of the surface layer.^106^, ^161^, ^164^

Discrepancies in elasticity measurements may also be resulted from the testing methods being used. Any method has a systematic error, such as the nonlinearity error in bending tests, substrate and confinement effects in nanoindentation tests,^31^, ^77^ uncertainty in boundary conditions for resonant vibration tests,^170^, ^242^, ^243^, ^244^ and EBI‐induced effects for in situ EM tests.^138^, ^142^, ^146^, ^147^, ^148^ In addition, misalignment can significantly affect the measured mechanical properties. In tensile testing, misalignment of the NW axis with loading direction is unavoidable due to the difficulties associated with specimen manipulation and clamping at small scale.^245^ In situ EM study on Au NWs showed that small misalignment could change the fracture mode from ductile to brittle.^226^ For bulk materials, the elastic strain obtained from bending is usually much higher than that from elongation, owing to their different stress distribution and sensitivity to defects and flaws. Such a discrepancy is expected to be much smaller in the testing of NWs, but cannot be neglected.^101^, ^108^

Discrepancies existing between simulations and measurements should be attributed to the inconsistent use of constitutive models, such as the models for the surface bond saturation or reconstruction, as well as the nonlinearity in elasticity.^184^, ^246^ Surface effects, contamination, and microstructural defects have been considered in some simulation studies, but the models for describing those phenomena are rather simple and assumptive. In atomistic simulations, the number of atoms used has limitations in order to reduce computation time. Thus the NWs being modelled usually have a size smaller than 20 nm, which is far smaller than those being measured in practical tests.

### 3.2 Anelasticity of NWs

Some bulk materials exhibit a time‐dependent elastic behavior during unloading, i.e. the deformation is fully recovered after a while when the load is completed released.^247^ Such a time‐dependent behavior is usually known as anelasticity or viscoelasticity. Nanomaterials can exhibit a much greater time‐dependent elasticity than their bulk counterparts, due to the coupling effects from their surfaces, interfaces and defects.^248^ In nanocrystalline metallic materials, anelasticity can be frequently observed, due to the cooperative motion of atoms in grain boundaries.^249^

Anelasticity in NWs may involve some different mechanisms, depending on the microstructure of specific NWs being tested. In single‐crystalline GaAs NWs, their anelasticity can be attributed to the existence of the amorphous surface oxide layer formed during sample preparation.^103^ When external stress is removed, the amorphous layer holds back the recovery of the crystal core, resulting in a delayed recovery. It is reasonable to infer that this type of anelasticity depends on the NW diameter because a thicker surface amorphous layer would cause a much higher recovery driving force in comparison to that generated by a thinner layer, hence a great anelasticity. It is speculated that the anelasticity in twinned CuO NWs might be related to the motion of atoms in the vicinity of twin boundaries.^129^ Under a large external bending stress, atoms adjacent to a twin boundary would move away from their original sites to new positions to counteract local lattice distortion. Once the external stress is removed, the rearranged atoms tend to move back to their original sites motivated by the global lattice distortion stress. Stress relaxation, however, cannot be achieved immediately when the temperature is low, resulting in anelastic deformation. Twin boundaries can block the motion of dislocations, preventing the NWs from fracture and facilitating anelasticity.

**Figure 21:**
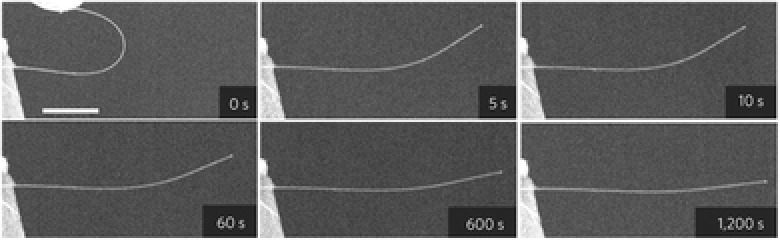
A sequence of SEM images showing the recovery process for a ZnO NW after removing the bending load. Scale bar, 2 µm. Reproduced with permission.^250^ Copyright 2015, Nature publishing group.

Single‐crystaline ZnO and p‐doped Si NWs exhibit a large anelasticity that is up to four orders of the greatest value observed in bulk materials, with a timescale in the order of minutes.^250^ The anelasticity of ZnO NWs cannot be explained using the oxide layer model^103^ or the twin boundary motion mechanism.^129^ As the relaxation strength (amplitude) is much greater, and the relaxation time of ZnO NWs is much longer than those respective values predicted using the thermoelastic relaxation mechanism, it is suggested that the anelasticity of ZnO should be controlled by the Gorsky relaxation,^251^ which arises from the motion of point defects in an inhomogeneous stress field. Nevertheless, the classic Gorsky relaxation predicts a linear relationship between the maximum anelastic strain and the initial strain, and has only been observed in a few bulk materials with very small relaxation amplitudes. Therefore, the expanded Gorsky relaxation model is proposed to interpret the nonlinear relationship when strain is great.^250^ According to this model, the ultrahigh bending stress (strain) applied to a NW is responsible for the great anelastic strain shown in **Figure** **21**. The diffusional flux of point defects depends on chemical potential gradient, which in turn affects the stress gradient. The small diffusion distance (due to the small NW diameter), high stress gradient and great diffusivity therefore lead to the relaxation (recovery) time in the order of minutes.

In general, the anelasticity of NWs should be attributed to the significantly strong surface and size effects. However, the underlying mechanisms are far from clear yet. The detailed diffusion pathways of the point defects for initial plastic deformation are unclear and the ensuing anelastic relaxation still remains as an open question. The effect of NW size on its anelasticity demands for further clarification.^252^ In addition, the current study of anelasticity of NWs relies on in situ EM techniques, but some preliminary results have demonstrated that EBI could speed up the elastic recovery of strained NWs. Therefore, new questions arise, i.e. whether EBI can induce anelasticity of NWs and how to determine the effect of EBI on anelasticity.^129^ This makes the study more complicated as the EBI effects involve the knock‐on effect on strained lattice atoms and the activation effect of diffusion of point defects in the strained NWs. *Ex situ* EM techniques, such AFM‐based^11^, ^22^ or OM‐based techniques,^145^, ^146^ can also be used for the comparative study of the anelasticity of NWs, in order to avoid the effects from electron beam irradiation.

Although the underlying mechanism remains unclear, the anelastic strain observed in the NWs was significantly higher than that for their bulk counterparts. This suggests that NWs could serve as highly efficient damping materials for vibration reduction on various applications such as manufacture of high precision machines and cutting tools.

### 3.3 Plasticity of NWs

Plasticity of a bulk material is often associated with the initiation and movement of dislocations, as well as the interactions between dislocations and grain boundaries, while plasticity of a nanocrystalline material is typically related to rotation of grains, dislocation‐grain boundary interaction and twin formation and rotation.^84^, ^253^, ^254^ Plasticity of NWs might even be different from those of bulk and nanocrystalline materials, due to their significant surface, interface and size effects.^246^, ^255^

Size‐dependent plastic deformations were frequently observed in various metallic NWs, such as Au,^226^, ^256^ Cu,^257^ Ag^117^ and Ni.^119^ Seo et al. found that defect‐free Au NWs of ≈100 nm in diameter exhibited super‐plasticity induced by the formation and propagation of partial dislocations.^90^ Subsequent TEM and electron backscattered diffraction analyses further revealed that single‐crystalline Au NWs of diameters of 40–200 nm had (partial) twinning‐mediated deformation patterns and a large number of small twins or a long twin being formed along the NWs.^258^ Such twinning‐mediated deformation was observed in the Au NWs of below 20 nm too.^226^ The plastic deformation of the Au NWs is apparently mediated by partial dislocations. In situ TEM demonstrated that the plastic deformation of Au NWs of below 10 nm in diameter is dominated by partial dislocations emitted from the free surface,^259^ while the plastic deformation of Au nanocrystal of 6 and 3 nm in particle size seems to be controlled by lattice slip.^260^, ^261^ Apparently the plastic deformation patterns are influenced by the geometric size of Au NWs. The size‐dependent plasticity of Au NWs observed in the experiments agrees well with that predicted by atomistic simulations, suggesting that atomistic simulations are valuable for understanding the nanoscale plasticity.^246^, ^262^

**Figure 22:**
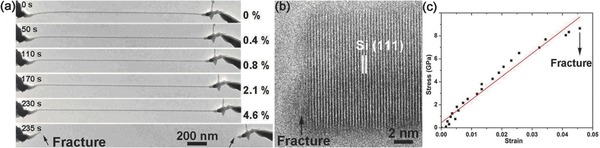
In situ TEM tensile test of a Si NW. Reproduced with permission.^101^ Copyright 2012, ACS. a) Snapshots of the tensile elongation and fracture process. b) An HRTEM image of the Si NW fracture surface. c) Typical stress‐strain curve showing a linear elastic deformation until abrupt failure with the values of fracture stress and strain of 8.7 GPa and 4.6%, respectively.

**Figure 23:**
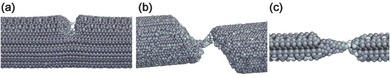
Snapshots of [110]‐oriented Si NWs under tension in molecular dynamics simulations using the modified embedded atom method potential. Reproduced with permission.^266^ Copyright 2007, Taylor&Francis. a) Brittle fracture of a 7‐nm‐diameter NW at 300 K. b) Ductile fracture of the same NW at 1000 K. c) Ductile fracture of a 2‐nm diameter NW at temperatures as low as 100 K.

**Figure 24:**
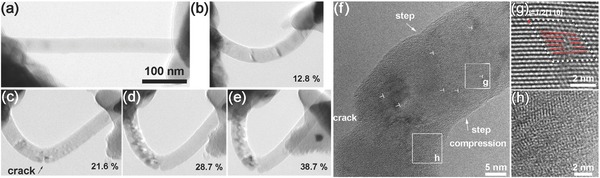
In situ TEM bending test on a single Si NW with a diameter of 25.3 nm. a‐e) TEM images of the bending process. b) Strain contrast was observed at a low strain. c) The crack was initialized at a strain of 21.6%. d, e) Under a higher bending strain, the crack propagated. f) HRTEM image of the Si NW in (d). On the tensed side, a crack was identified. On the compressed side, the structure became amorphous. Dislocations were observed around the severe deformation zone, and steps were found on the surface. g, h) HRTEM images of dislocation and the amorphisized area. Reproduced with permission.^101^

There exist significant discrepancies on the plasticity of semiconductor NWs between experiments and simulations. Nanoscale compression of the FIB‐fabricated Si pillars with diameters ranging from 230 to 940 nm demonstrated that brittle‐to‐ductile transition occurred, when the pillar diameter was between 310 to 400 nm.^263^ Some early In situ TEM bending and tensile tests indicated that Si NWs with diameters of from 60 to 100 nm exhibited large‐strain plasticity at room temperature, due to the extensive dislocation activities and crystal‐to‐amorphous phase transformation.^102^, ^264^ However, recent AFM‐based tests,^11^, ^22^, ^31^ in situ SEM^86^, ^88^, ^265^ and in situ TEM^101^ tests showed that Si NWs had only brittle fracture. As shown in **Figure** **22**, the fractured surface of Si NWs with diameters down to 5 nm shows no evidence of plasticity, which agrees well with the linear stress‐strain curve until NW's breaking and the MD‐simulated results that brittle fracture occurred in the 5 nm Si NWs.^101^ In addition, this is also consistent with the previous MD simulations that Si NWs of above 5 nm in diameter still fracture in a brittle mode at room temperature, while brittle‐to‐ductile transition only occurs in Si NW of above 5 nm in diameter at elevated temperatures or in small NWs of below 4 nm in diameter (see **Figure** **23**).^266^ As the same Si NWs exhibited considerable plastic deformation and crystalline‐amorphous transition in the in situ TEM bending test, the testing methods and conditions were presumed to play critical roles in the mechanical behaviors of NWs, as shown in **Figure** **24**.^101^ Difference and controversy on the size‐dependent brittle‐to‐ductile transition can also be found in ZnO NWs. Atomistic simulations suggest that ZnO NW with diameters above 5 nm have no brittle‐to‐ductile at room temperature. However, crystalline‐to‐amorphous transformation has been observed in the bent ZnO NWs with diameters up to 110 nm.^195^

The size‐dependent brittle‐to‐ductile transition in ceramics at room temperature was well demonstrated using compression testing of FIB‐fabricated SiC pillars with diameters down to submicron.^267^ In situ EM bending and tensile tests found that brittle‐to‐ductile transition occurred inside SiC NWs when experienced a strain of ∼2%, thus allowed superplastic deformation of a local strain of exceeding 200%. However, an early AFM bending test showed that single‐crystalline SiC nanorods fractured in a brittle mode, and a recent in situ SEM tensile test^233^ also found that SiC NWs with diameters down to 26 nm still deformed elastically until brittle fracture, exhibiting no evidence of plasticity. The loop testing of SiC NWs showed that the NWs with diameters down to 29 nm fractured in a brittle mode at room temperature in an ambient atmosphere, but EBI could induce crystal‐to‐amorphous phase transition in the strained NWs.^145^

The size‐dependent plastic deformation has be frequently observed in the mechanical characterization of NWs. The differences exist in the previous experimental and simulated results should be attributed to a number of factors, as summarized below. *(i) Strain rate*. The MD simulations of plasticity is conducted at strain rates of 10 to 15 orders of magnitude higher than experimental ones due to computational limitations. Both experimental tests^268^ and atomistic simulations^269^ showed that brittle‐to‐ductile transition is sensitive to strain rate. Therefore, stain rate is one of the important factors responsible for the differences observed in experimental tests, as well as the discrepancies between experimental and simulated results, such as the brittle‐to‐ductile transition in Si NWs.^22^, ^101^, ^264^, ^266^ *(ii) Surface contamination or amorphous layer*. Most experimental tests and atomistic simulations have not considered the effects of the amorphous oxide layer on the deformation behaviors of semiconductor NWs. This should be another important cause for the difference between experiment and simulation, as simulations are carried out in an ideal situation. Recently, it has been proved experimentally the surface amorphous layer introduced during the FIBed process of Si crystal is the real reason for what so called brittle‐to‐ductile transition of FIBed Si pillars.^270^ In addition, it was further demonstrated that by taking advantage of the confinement effect of the surface soft amorphous Si layer, deformation‐induced crystalline‐to‐amorphous transition mechanism can be revealed.^82^ This finding also paves the way to study the plastic deformation mechanism of brittle materials inside TEM. In fact, some of simulation studies started to investigate the effect of amorphous surface layer on brittle‐to‐ductile transition of SiC NWs.^204^, ^271^ *(iii) Loading conditions*. The brittle‐to‐ductile transition in brittle NWs is relatively easy to detect under bending or compression, in comparison with tensile testing.^101^ Misalignment in tensile tests could also change the deformation behaviors of NWs.^226^ *(iv) Microstructures*. Depending on the specific fabrication process, NWs of the same material but used in different tests might have differences in microstructural features such as defects and growth directions, and such differences are difficult to be determined precisely. For example, MD simulation revealed that defects in SiC NWs might lead to brittle‐to‐ductile transition during tensile testing. This suggests that the different deformation behaviors observed in the experimental characterization of SiC NWs might be attributed to their microstructural difference.^204^ Recent comparative in situ EM tensile tests demonstrated that five‐twinned silver NWs underwent stress relaxation during loading and then completed plastic strain recovery during unloading, while the other similar experiment on single‐crystalline silver NWs could not show such a behavior.^117^ It is presumed that vacancies can reduce dislocation nucleation barrier, facilitating stress relaxation, while twin boundaries and their intrinsic stress field may promote retraction of partial dislocations. *(v) Testing environments*. Unlike the macroscale mechanical characterizations, the observed mechanical properties of NWs may strongly depend on the specific testing environment, such as EBI, laser‐beam irradiation and ambient atmosphere. For example, the size‐dependent deformation of NWs was mainly observed in the in situ EM examinations, but the effect of EBI on the deformation of NWs has not been studied systematically till now. Currently, the effect of EBI is attributed to TDE and sample heating. However, sub‐threshold irradiation may also lead to a significant microstructural evolution, which could be done through promoting point defect diffusion and enhancing dislocation loop growth in ceramics and semiconductors.^136^ Under mechanical loading, the activation energies for the generation and diffusion of point defects and dislocations in a strained NW are expected to be much less than that for a strain‐free material. Therefore, whether and how the sub‐threshold EBI in SEM/TEM affects the deformation behaviors of NWs requires comprehensive understanding.^83^, ^84^ In addition, it is well known that the ambient atmosphere can change or modify the surface structures of some materials, such as H_2_ on Si surfaces and H_2_O on ZnO surfaces, and thus change the mechanical properties of these surfaces. These will change the mechanical properties of the corresponding NW materials due to their large surface‐to‐volume ratio, as predicted by some theoretical calculations and simulations.^186^ In the three‐point AFM bending measurement of ZnO NWs, Yong et al. found that the elastic modulus of the NWs increased significantly from 40 GPa to 88 GPa as the increase of the ambient humidity from 50% to 80%.^197^ First‐principles density functional theory calculations suggested that the water molecules adsorbed on the ZnO surface would attract surface Zn atoms to move outward and hence increase the value of surface stress of ZnO surface.^197^

### 3.4 Strength of NWs

The yield or fracture strength of a bulk material is significantly lower than the respective theoretical values predicted using the atomic‐bonding energy, due to the existence of defects or flaws in the material. The simplest way to strengthen a material is reducing defects or flaws in the material or making the material smaller that thus reduces the defects. This can be easily understood from the Griffith's famous prediction in 1921 that “in the limit, in fact, a fiber consisting of a single line of molecules must possess the theoretical molecular tensile strength”.^272^ NWs have quite limited defects, and thus usually exhibit ultrahigh strengths, some of which are close to their theoretical values, as can be seen from Table 1. Therefore, the testing of mechanical strength of NWs can provide not only a direct approach to validate theoretical calculations, but also a test platform for quantitatively understanding the effects of size, surface and defect on the strength of materials, thus proving that “smaller is stronger”. Theoretically, the binding energy of surface atoms is somehow different from that of bulk atoms due to the effects of surface reconstruction and relaxation, which may alter the fracture stain and strength of a surface atomic layer. As a result, the surface effect can strengthen or weaken a specific NW due to its increased surface‐to‐volume ratio.^107^, ^180^ As the strength is usually very sensitive to unavoidable defects in NWs, it has been very challenging to distinguish or isolate the contribution from such a surface effect.

The effects of planar defects on the strength of NWs have attracted great interest very recently. Jang et al. investigated the effects of diameter, twin‐boundary spacing and twin‐boundary orientation of Cu nanopillars with a very high density of periodic twin boundaries on the mechanical responses, using in situ TEM tensile test and atomistic simulations.^122^ They showed that the tensile stress of a pillar of 100 nm in diameter with orthogonal twin boundaries spaced at 4.3 nm ranged from 1.81 to 2.45 GPa, giving an average value of 2.12 GPa.^122^ This value is ≈ 40% of the intrinsic strength of Cu^228^ and 1.5 times higher than the ultimate tensile strength of single‐crystalline Cu nanopillars with similar diameters tested using an identical methodology.^273^ In situ TEM compression tests also showed that Cu pillars with diameters of 100 nm with orthogonal twin boundaries exhibited a ductile‐to‐brittle transition caused by the reduced twin boundary spacing.^122^ The corresponding atomistic simulations showed that such brittle fracture might be associated with the intrinsic brittleness of twin boundaries.^122^ It is well documented that fcc metallic NWs usually have a five‐twinned structure with twin boundaries parallel to the axial direction. In this case, twin boundaries might effectively block dislocation motion during bending, and thus strengthen the twinned NWs.^274^ As a result, it was shown that five‐twinned Ag NWs exhibited an extremely high yield strength of 7.3 GPa in the bending test.^46^ This is much higher than the maximum yield strength of 2.64 GPa and ultimate tensile strength of 4.84 GPa observed by in situ SEM tensile test, Ag,^91^ 2.94 GPa by in situ TEM tensile test,^275^ and also higher than the theoretical tensile strength of 3.5 GPa for the 110‐oriented Ag single crystal calculated using the Schmid factor analysis.^276^

Unlike the strengthening effect of twins on metallic nanopillars, collapse could take place in SiC NWs in the regions with periodic 90° nanotwins (i.e. twining plane perpendicular to the growth direction).^277^ The systematic MD simulation on SiC NWs indicated that the pure 3C‐structure of SiC was the strongest with a strength of 28.5 GPa, the highly defective structure was the second strongest, and the 3C structure with 19.47° stacking faults was the weakest.^204^ This agrees well with the in situ SEM tensile test of SiC NWs, which revealed that cracks always initiated and propagated in the 3C segments with 19.47° stacking faults, giving a maximum tensile strength of 25.3 GPa.^233^ The loop bending test of SiC NWs also indicated that cracks were initiated at the ends of the stacking faults on NW surface, giving an elastic strain of 4 to 7% and average strength of 27.5 ± 7.5 GPa.^145^ The measured strength values of SiC NWs with stacking faults are much lower than the theoretical prediction of 53.4 GPa for single crystal SiC. This suggests that twins and stacking faults decreased the strength of SiC NWs, supported by the previous simulation.^204^ Nevertheless, it is still unclear that whether or not such weakening effect of stacking faults prevails in other ceramic NWs. Planar defects strengthened the brittle semiconductor GaAs NWs.^104^ The GaAs NWs with a perfect single crystal structure could experience an ultimate compressive stress of 6.2 GPa, which agrees with the value of 6.4 GPa predicted by MD simulation, but the GaAs NWs with a high density of planar defects exhibited a much high strength of 9.2 GPa.^104^ As the GaAs NWs with and without defects had the same sizes of ≈ 60 nm in diameter and were tested using the same set‐up and testing conditions, it appears convincing that the high density planar defects might hinder crack initiation and thus strengthen the GaAs NW.^104^

**Figure 25:**
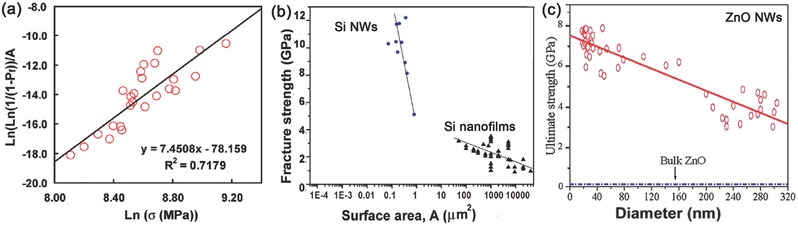
a) Fracture strength obtained from in situ TEM tensile test plotted against the surface area of ZnO NWs. Reproduced with permission. Reproduced with permission.^108^ Copyright 2009, ACS. b) Fracture strength obtained from in situ SEM tensile test plotted against the side surface area of Si NWs and nanofilms. Reproduced with permission.^86^ Copyright 2009, ACS. c) Fracture strength obtained from AFM three‐point bending test plotted as against the diameter of ZnO NWs. Reproduced with permission.^49^ Copyright 2008, APS.

Agrawal et al. developed a probabilistic model to examine the relationship between the fracture strength and surface area of NWs.^108^ According to the Weibull statistics theory, the probability of failure *P*_τ_ for a specimen of surface area *A* under uniaxial stress can be written as,^108^

(23) $$P_{\tau }=1-\exp\left(-{\left(\sigma_{f}/\sigma_{oA}\right)}^{m}A\right)$$

where σ_*f*_ is the failure strength and σ_*oA*_ is the characteristic strength relative to unit volume or surface area, and *m* is the Weibull modulus. It was found that the measured fracture strength of ZnO NWs matched well with the Weibull statistics, as shown in **Figure** **25**a. However, He et al. could not fit the strength data of ZnO NWs obtained from the in situ SEM tensile test by use of Equation (23).^278^ Zhu et al. showed that σ_*f*_ of Si NWs logarithmically related to the surface area in Figure 25b.^86^ A linear relationship between fracture strength and diameter was found in AFM 3‐point bending of ZnO NWs, as shown in Figure 25c.^49^ Similar linear relation was also found in the Si NWs measured using AFM bending.^22^ Apparently there is a clear size dependency on the strength of NWs, but there appears lacking of a universal relationship between the strength and surface area.

**Figure 26:**
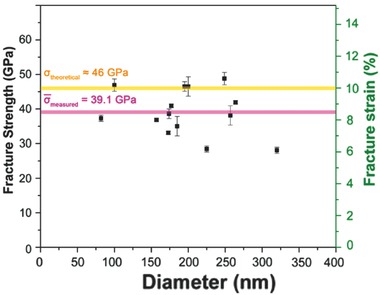
Fracture strength obtained from a loop bending test plotted against the diameter of α‐Al_2_O_3_ NWs. Reproduced with permission.^146^ Copyright 2013, IOPscience.

It is extremely challenging to quantitatively characterize point and surface defects of NWs, so establishment of the relationship between mechanical strength and size of NWs might be infeasible at this stage. Nevertheless, the nanoscale volume and surface of a NW implies significant decrease in defects in the material. Using NWs as a testing vehicle, experimental measurement of the intrinsic strength of a material becomes feasible. Also, the selection of testing methodology is important as the loaded region in bending, especially two‐point bending based on the pure‐bending model, is much smaller than that in a tensile test, which can significantly reduce the effect of defects, hence size. Under such circumstance, the strength obtained should be independent of the size of NWs. **Figure** **26** shows the fracture strength of α‐Al_2_O_3_ NWs being measured using a loop bending test,^146^ where the strength data appear to be irrelevant to the NW diameter and are very close to the theoretical strength predicted by the first principles calculation. Similar results were also obtained in the mechanical characterization of ZnO,^23^, ^200^ Si,^11^, ^265^ GaAs^104^ and SiC NWs.^145^

As for amorphous NW materials like metallic glasses, although they do not possess any structural defects such as dislocations or grain boundaries, strong size effects on the strength have been frequently observed.^279^, ^280^ It was previously shown that the size effect might be involved with the irradiation damage induced by the Ga ion bombardment during the FIB sample preparation.^123^, ^281^ However, a recent in‐situ TEM measurement of glassy Pd_40_Cu_30_Ni_10_P_20_ NWs, which were not prepared by FIB, revealed that the tensile strength and elastic strain the NWs deceases from 2.8 to 1.75 GPa and from 3.2 to 5%, respectively, as the increase of NW diameter from 340 to 1230 nm.^282^ Therefore, it was suggested that “*smaller is stronger*” should be still valid for amorphous NWs, due to the transformation of deformation mode at critical size.^279^, ^283^

## 4 Concluding Remarks

Characterizing the mechanical properties of NWs has been a daunting task for researchers due to a poor understanding of the deformation mechanics and the difficulties encountered in the manipulation and control of specimens during mechanical testing. In current literature, the obtained mechanical properties can sometimes be contradictory, and are often substantially lower than their respective theoretical predictions. It remains exceptionally challenging to accurately characterize the mechanical properties of NWs due to the lack of a comprehensive understanding of their intrinsic and size‐dependent behavior. The advance in nanomechanical testing methods in recent years, especially in situ EM techniques, has enabled significant progress towards an improved understanding of NW mechanical behavior, including deformation physics, elasticity, plasticity and strength. In particular, the unique properties of a solid material are not commonly observed in conventional bulk materials, including superelasticity, superplasticity, recoverable plasticity, anelasticity, and ultrahigh strength, have been discovered during the testing of NWs. Consequently, the study of NWs over the past two decades has significantly advanced our knowledge of materials.

Among current methodologies, in situ EM testing is widely accepted as the most effective solution for the characterization of NWs. This is attributed to high‐resolution loading and positioning of specimens, the ability to monitor the deformation process in situ, and conduct post‐deformation analysis. Nevertheless, the initiation and accelerated movement of defects in a specimen due to EBI, must be carefully considered, especially if the specimen in under strain. For this reason, a systematic study of the effects of EBI on the mechanical behavior of a specimen during in situ EM characterization is essential. AFM, nanoindentation, and optical microscope based techniques are therefore indispensable complimentary methodologies for developing a complete understanding of the mechanical properties of NWs.

The surface effects play an important role in the mechanical behaviors of NWs, with the capacity to enhance or diminish their mechanical properties. The surface effects on the plasticity and strength of NWs requires further clarification; many in situ TEM studies indicate that dislocations easily nucleate at the free surface of strained NWs. The size effect on the mechanical property of NWs is a consequence of reducing the number of defects within its volume. It remains tremendously challenging to quantitatively evaluate defects within a NW, especially point defects Therefore, an improved understanding of the relationship between surface and size effects, defects, and the mechanical properties of a NW will remain a significant focus of future research.

The majority of current studies involved in characterizing the mechanical properties of NW do not substantially explore how the testing environment affects the obtained results. As a primary building block of the future generation of nanodevices, NWs will be exposed to a range of environmental conditions. Several studies have shown that humidity, surface oxidation, and surface contamination may have a significant effect on NW elasticity, as well as fracture behavior. Temperature is another important environmental factor. The temperature dependency of the mechanical properties of NWs is expected to differ from that of their bulk counterparts. Overall, how the mechanical properties of NWs are affected by their environmental conditions, including temperature, humidity, and the potential for contamination, needs to be studied systematically. Such studies would be of great scientific importance for the advancement of materials science, and for the future development of nanodevices.
